# Network methods to support user involvement in qualitative data analyses: an introduction to Participatory Theme Elicitation

**DOI:** 10.1186/s13063-017-2289-5

**Published:** 2017-11-23

**Authors:** Paul Best, Jennifer Badham, Rekesh Corepal, Roisin F. O’Neill, Mark A. Tully, Frank Kee, Ruth F. Hunter

**Affiliations:** 1 School of Social Sciences, Education and Social Work, Queen’s University, Belfast, UK; 2 School of Medicine, Dentistry and Biomedical Sciences, Queen’s University, Belfast, UK

**Keywords:** Network analysis, Participatory analysis, User involvement, Trials, Patient and public involvement

## Abstract

**Background:**

While Patient and Public Involvement (PPI) is encouraged throughout the research process, engagement is typically limited to intervention design and post-analysis stages. There are few approaches to participatory data analyses within complex health interventions.

**Methods:**

Using qualitative data from a feasibility randomised controlled trial (RCT), this proof-of-concept study tests the value of a new approach to participatory data analysis called Participatory Theme Elicitation (PTE). Forty excerpts were given to eight members of a youth advisory PPI panel to sort into piles based on their perception of related thematic content. Using algorithms to detect communities in networks, excerpts were then assigned to a thematic cluster that combined the panel members’ perspectives. Network analysis techniques were also used to identify key excerpts in each grouping that were then further explored qualitatively.

**Results:**

While PTE analysis was, for the most part, consistent with the researcher-led analysis, young people also identified new emerging thematic content.

**Conclusions:**

PTE appears promising for encouraging user led identification of themes arising from qualitative data collected during complex interventions. Further work is required to validate and extend this method.

**Trial registration:**

ClinicalTrials.gov, ID: NCT02455986. Retrospectively Registered on 21 May 2015.

## Background

With research funding imperatives surrounding user involvement now well established [1, 2], the coproduction of research has gained significant momentum in recent years [3–5]. A ‘coproduction’ model typically requires end users to become ‘co-researchers’ and thus full and equitable members of research teams [6]. Despite this commitment, the challenge of involving end users in *meaningful* ways remains difficult, particularly in regard to health and social care research. Often viewed as tokenistic with little consideration of the power dimensions at play [7, 8], participatory approaches have struggled to fully establish methodological rigour in this area.

Given their complexity, it could be argued that randomised controlled trials (RCTs) will benefit most from increased user involvement [9]. However, this is not reflected within the published academic literature whereby the quality of Patient and Public Involvement (PPI) outcomes is inconsistency reported [10]. Dudley and colleagues found that training was a particular issue (both for researchers and members of the public) [11]. There were concerns that too much training could professionalise the role of the user representative and dilute the ‘patient perspective’.

The challenge of meaningful engagement is no more apparent than when user involvement is examined in data analyses and interpretation [12]. Byrne et al. [13] note that ‘while there are growing examples of consulting with and actively involving research participants in the planning, designing and data collection phases of a project, examples are few of participatory interpretation and analysis of data’. While some attempts have been made to ‘democratise’ the data analysis process in research with both adults [6] and children [13–15] the adoption of these approaches has been far from widespread or adequate. Consequently, there is a pressing need for reliable, structured and accessible methods of involving end users in data analyses – particularly, for those involved in RCTs where the focus is often on quantifiable outcomes and the effectiveness of the intervention being trialled.

There are some aspects of data analysis where participatory methods are available. For example, Sweeney et al. [16] engaged lay researchers in coding of qualitative data. However, it is difficult to see past the power imbalances at play with the scientific researcher remaining in control throughout the process. Moreover, Garfield et al. [17, 18] note a general lack of infrastructure to facilitate meaningful involvement by service users. In an effort to address some of these issues, the Voice Relational Method was developed by Byrne and colleagues [13] whereby users reviewed transcripts (alongside the research team) and developed their own narrative regarding the emerging content. While promising, the feasibility of the approach may be in doubt when resources are constrained, given the significant training required by ‘lay researchers’. In order to get to a stage where research is conducted ‘with’ or ‘by’ (opposed to ‘on’) users, the barriers created through a lack of adequate training must be removed [19].

Card-sorting methods address a different aspect of analysis, namely organising knowledge. Participants sorting items into groups is an effective way to access different understandings of important dimensions of knowledge [20] and has been used in fields as diverse as menu structures for computer applications [21] and classifying features of behaviour-change techniques [22]. When embedded in a process of brainstorming and prioritisation, sorting is also used for participatory public health planning (Burke et al. [23], referred to as Concept Mapping in this context).

Involving children and young people as co-researchers adds an additional level of complexity as power imbalances are inevitable. Nonetheless, as young people’s lives today are so complex some sort of ‘insider’ knowledge is required to assist the researcher in understanding their data [24]. Kirby [25] supports this view by suggesting that young people may identify issues often overlooked by others. This is particularly important for health interventions delivered through RCTs which are often complex. Further benefits include a greater understanding of the views of young people and assisting with the prioritisation of agendas in policy and practice [15]. Within health and social care research, youth advisory panels appear to be one of the most popular methods of achieving Patient and Public Involvement (PPI). Morgan et al. [26] note how PPI groups can play an important role in terms of engaging ‘hard-to-reach’ groups. Yet, the extent to which these panels fulfil more than a rubber-stamping process remains to be seen. To date, there is little in the way of specific methods or standardised approaches for this aspect of research. This leaves the ‘door open’ for tokenistic engagement.

In this study, we propose a new approach to participatory data analyses called Participatory Theme Elicitation (PTE). Designed for qualitative data, the main task of PTE is to identify common groupings or ‘themes’ present in the data as recognised (in this instance) by a group of young co-researchers. It is important to note at this point that qualitative research and approaches to user involvement (e.g. PPI) are two distinct entities [26]. Qualitative research is well establish and defined in its approach, whereas user involvement is less so and focusses more on the process of participation within research rather than aligning itself with a particular method (e.g. qualitative or quantitative). PTE may offer a more considered approach to participatory data analyses and interpretation, particularly with children and young people as it empowers users and reduces researcher influence over the emerging thematic content. While related to existing sort methods, PTE uses network analysis techniques to construct the groupings and highlight patterns in the data for further qualitative exploration, which stimulates a different perspective on the data. The aim of this ‘proof-of-concept’ study was to test the feasibility and process of PTE, supplementing the primary researcher-led qualitative analysis conducted as part of the StepSmart Challenge of a school-based physical activity intervention [27]. Specific objectives of this study included:

- To develop a short training programme for young people (co-researchers) regarding the basics of qualitative data collection and analysis
- To test and refine data selection and sorting procedures
- To assess the suitability of network analysis community detection algorithms for identifying themes in the data; and
- To refine the protocol for PTE for further development and testing of the methodology

### Context: the StepSmart Challenge

The qualitative data used within this paper were gathered as part of the StepSmart Challenge feasibility RCT (ClinicalTrials.gov, Trial Number: NCT02455986). The StepSmart Challenge was a 24-week, school-based, physical activity intervention. The aim was to test the feasibility of an intervention targeting an increase in physical activity among 12–14-year-olds using a competition-based format. The competition took place on a school- and team-based level and contained intervention components, such as goal setting, prompts, self-monitoring and habit formation, which fit within a Self-regulation Control Theory framework [28]. Additional techniques, such as motivational messages (persuasion) and social support (vicarious experience), were used to influence self-efficacy perceptions according to Social Cognitive Theory [29]. Self Determination Theory [30] also played a key role in the development of the intervention, with the gradual withdrawal of material incentives and rewards (prizes, reward badges) as a means of encouraging a shift from extrinsic to intrinsically motivated physical activity behaviour [31].

Focus group interviews during the StepSmart Challenge were conducted across three different time points (baseline, 8 weeks and 24 weeks) within each intervention school (*n* = 3). The same individuals were longitudinally followed up with the number of respondents ranging from 19 to 27. Focus group interviews lasted from 24 to 40 min depending on numbers and availability of pupils within the school timetable. As such, nine transcripts were generated.

## Methods

The methods that we describe within the current study focus on the PTE elements only, though we contrast the findings to those from a researcher-led analysis within the discussion. Focus group data collected as part of the StepSmart Challenge was analysed using a thematic framework [32] by two members of the research team (RC, PB). To maintain their independence, the *data selection* component of PTE (see below) was conducted before thematic analyses was undertaken and neither researcher was made aware of the PTE groupings until after their analysis was complete. Due to practical considerations, it was not possible to include all of the transcribed focus group data as part of the PTE. However, details regarding the separate thematic analysis (using the entire dataset) can be found elsewhere [24].

PTE organises qualitative data by: (1) asking users to sort information into groups that are similar and individually meaningful to themselves and (2) the researcher(s) subsequently creating an overall set of groups that preserves as much as possible of the individual groupings. This process assists users to undertake the sorting task, and uses the results to stimulate further discussion.

Research methods were designed to serve the conventional approaches to analysis used within the StepSmart Challenge, with modifications to support supplementary PTE elements as appropriate. The sorting task was performed by the PPI panel used to evaluate the project. Researchers then used network analysis techniques to interpret the created groups, but final reflection and discussion with users was not undertaken in this proof-of-concept study.

### Recruitment

A convenience sampling approach was used to recruit co-researchers from two schools that had not taken part in the StepSmart Challenge [33]. These schools were identified using a pre-existing database of those who had engaged in research with Queen’s University, Belfast (Northern Ireland). From this, eight young people (aged 12–14 years) were invited to become members of a youth advisory panel (YAP) for the StepSmart Challenge. The YAP consisted of four male and four female young people and represented a mix of those who were both physically active and inactive, as informed through discussions with teaching staff. Prospective YAP members were given Information Sheets and Consent Forms before agreeing to take part and the study was granted ethical approval by the School of Medicine, Dentistry and Bio-medical Sciences Research Ethic Committee, Queen’s University, Belfast (REF: 15.09v3).

### Implementation of PTE

The implementation of PTE involved five key sequential steps: (1) capacity building; (2) data selection; (3) data sorting; (4) data grouping and (5) analysis.

#### Step 1: Capacity building

In order to build capacity, the research team met with YAP members on four separate occasions. Each meeting lasted between 90 and 120 min and included topics, such as how to design and conduct research; the importance of physical activity and the components of the StepSmart Challenge. Each session also included a number of interactive activities, e.g. short challenges or team games. The final session included a recap of areas covered, as well as some specific guidance regarding qualitative data analysis [19].

This final session began with a number of basic age-appropriate examples – using images rather than excerpts/quotes – to demonstrate the type of sorting techniques used during analysis of qualitative data. Discussions between teaching staff and research team members took place prior to the final session to ensure the acceptability of the content used. Firstly, the YAP members were independently provided with images of different foods and asked to sort these into groups by similarity; some young people categorised by food colour, others by shape, and others by food groups such as fruit or vegetable. In a second exercise we provided the YAP members with a series of images relating to different sports and asked them to also categorise these by similarity; some members grouped by team versus individual, others by the type of exercise, and others grouped by those requiring equipment versus no equipment. After completion of both sample exercises, discussions between panel members and the research team suggested that the YAP members were aware of the subjective nature of qualitative research and were confident with the sorting technique required for the main task. By increasing the panel’s knowledge of each of these key areas, the aim was to facilitate more informed decision-making during data analysis and interpretation of findings.

#### Step 2: Data selection

**Fig. 1:**
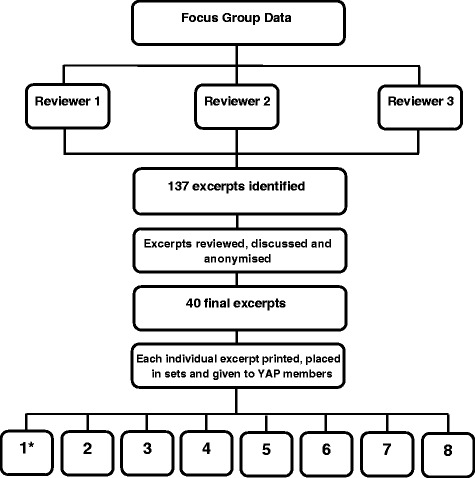
Preparation of focus group data. *Each box labelled 1–8 represents an individual youth advisory panel (YAP) member

Three members of the research team (PB, RC and RO’N) reviewed the nine transcripts from focus groups collected as part of an in-depth process evaluation of the StepSmart Challenge and identified excerpts that could be easily understood and interpreted as standalone statements. No formal coding of data had taken place prior to this activity and no conscious effort(s) were made to group data together or select excerpts based on potential significance. This produced an initial list of 137 excerpts (or quotes) taken across the three different time points (baseline, 8 weeks and 24 weeks). Further discussion among the research team members revised this number to 40 final excerpts (see Fig. 1). This decision was based on a number of factors, namely potential fatigue, readability of excerpts and repetition. All 40 excerpts were given an anonymous code so the research team could identify the relevant quote by school, gender and study time point. All qualitative excerpts were re-checked and anonymised before being presented to YAP members. The full set of excerpts used is provided in the Appendix.

#### Step 3: Data sorting

These 40 excerpts were then used as part of the data sorting phase of PTE; eight folders were created – one for each YAP member – containing the same 40 excerpts individually printed and labelled 1–40. Panel members worked independently over a 2-h period, initially spending time reading and re-reading in order to familiarise themselves with and make sense of the data provided in the 40 excerpts.

Panel members were instructed to sort the excerpts into piles, based on similarity, using whatever criteria they found relevant [20] with a minimum of two piles and no miscellaneous pile.

**Fig. 2:**
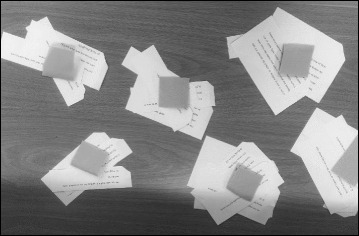
Sorting example

No further instructions were given, informed by previous research suggesting that over instruction may limit or constrain lay analyses to the point where one is never ‘surprised’ [18]. Data sorting was completed simultaneously by all YAP members as an independent process, without discussion about the choices they were making (see Fig. 2). Members of the research team were on standby to assist panel members if they had any questions and a PowerPoint slide containing task instructions was visible throughout.

After this process, each panel member had between 5 and 14 piles of similar excerpts. These piles were labelled numerically but in no particular pile order for the purposes of the next step. Piles from each young person were recorded and uploaded into a Microsoft Excel spreadsheet (see Table 1) by R’ON who was not involved in further analysis. Three columns were generated to distinguish (1) the young person; (2) the excerpt ID number and (3) the pile. For example, if young person 1 grouped excerpts ID1, ID2 and ID5 together this would be assigned a group label, such as Gp5. This process would continue until all excerpts in the set had been given a group label, thus enabling comparison across groupings. It is important to note, however, that the group label is used only to distinguish groups within individual sets and not across YAP members.

**Table 1:** Example of data imputation following grouping task

Participant ID	Excerpt identification number	Pile/Group label
P1^a^	ID1^b^	Gp5^c^
P1	ID2	Gp5
P1	ID3	Gp7
P1	ID4	Gp7
P1	ID5	Gp5
P2	ID1	Gp2
P2	ID2	Gp6
P2	ID3	Gp6
P2	ID4	Gp2
P2	ID5	Gp2
P3	ID1	Gp2
P3	ID2	Gp2
P3	ID3	Gp6
P3	ID4	Gp6
P3	ID5	Gp7

^a^ *P1* participant 1, ^b^ *ID1* excerpt 1, ^c^ *Gp5* grouping 5

#### Step 4: Data grouping

The next step of the PTE method combines the piles of individual YAP members into an overall set of piles and is known as ‘grouping’. PTE grouping was conducted by JB who was not involved in any prior analysis of qualitative data from StepSmart. It first uses the spreadsheet of assignments (see Table 1) to calculate a similarity score for each pair of excerpts. With 40 excerpts, there are 780 (40 × 39/2) potential pairs. For each pair, the similarity score is the number of YAP members who placed both quotes together in the same pile. In this case, there were eight sorters and hence a maximum score of 8 for each pair of excerpts.

The standard method for combining sort piles is to use multidimensional scaling to place each item in two dimensional space so that similar items are close together [34]. A spatial clustering technique is then used to allocate the items to an arbitrary number of groups, assigning items close to each other to the same group. However, projecting into two dimensions distorts the relationship between distance and similarity.

Instead, with PTE, the natural representation for entities and relationships is used, a network. Each excerpt was represented by a node (coloured circle in Fig. 4), with pairs of nodes connected by an (undirected) tie if at least one person placed the two excerpts in the same pile (that is, similarity score of 1 or more), and with the thickness of the line indicating the number of YAP members who have placed those excerpts into the same pile. The scores were converted into a (conceptual) distance between pairs of excerpts, calculated as 1/score. So, the maximum score of 8 returns the smallest conceptual distance of 0.125, and the minimum score of 1 returns the largest distance of 1. The conceptual distance provided a weight for each tie. Network analysis methods are then used to generate groups, and to assist with interpretation of these groups.

Excerpts were assigned to a thematic cluster using the cluster_edge_betweenness procedure in the igraph R package [35], which implements a well-established algorithm used to detect communities in social networks [36]. This algorithm relies on the network concept of ‘betweenness’, with high values indicating that the tie joins together groups of nodes (i.e. excerpts) that are fairly distinct from each other (in this case, conceptually distant). The algorithm removes the tie with the highest betweenness value iteratively, recalculating betweenness values after each removal, gradually fragmenting the network into separate communities.

**Fig. 3:**
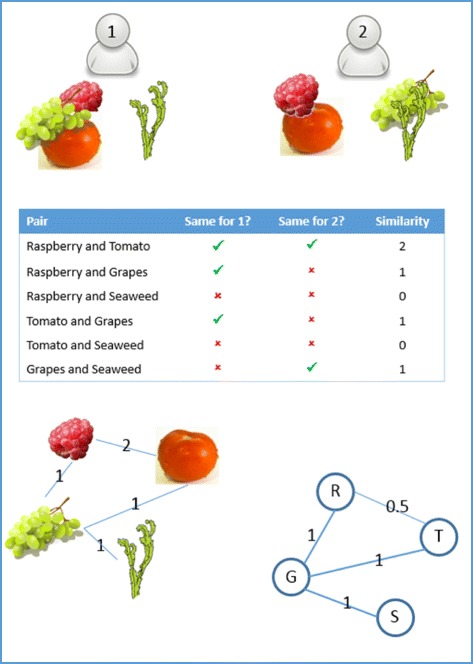
Constructing the network for grouping

In this hypothetical example (Fig. 3), person 1 has placed the raspberry, tomato and grapes in one pile and the seaweed in a different pile (perhaps based on ‘like to eat’). Person 2 has placed the raspberry and tomato in one pile and the grapes and seaweed in a separate pile (perhaps based on colour). The table shows the calculation of the similarity score for each pair of items. Those similarity scores are transferred to a network (with no edge where the score is zero), and the inverse of the scores is used for the edge weights of the final network (bottom right). If used on that final network, the community detection algorithm would find only a single group.

#### Step 5: Analysis of thematic groups

**Fig. 4:**
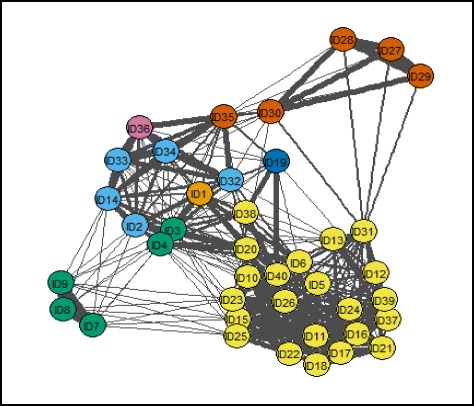
Network diagram of sorting results. Each circle represents a quote and a line between them indicates that at least one youth advisory panel (YAP) member placed the two excerpts into the same pile, with a thicker line where more YAP members did so. Circles with the same colours indicate that the relevant excerpts were assigned to the same theme, with three excerpts (ID1, ID19 and ID36) not belonging to any theme

For the purposes of this proof of concept, we used the network visualisation of the groups (Fig. 4) to stimulate researcher consideration of the identified themes. This was informed by network analysis techniques to identify the quote in each cluster for which the average weighted distance to all other quotes was lowest (referred to as ‘closeness centrality’) and the quote that is best placed to bridge the conceptual distances (referred to as ‘betweenness centrality’). A thematic interpretation, informed by the principles of thematic analysis [29], was then applied to qualitatively explore the underlying meaning contained within each grouping and excerpt.

## Results

PTE analysis identified seven unique groupings (see Fig. 4). The excerpts within each of the seven groupings were then reviewed by the authors for thematic patterns. Four core groupings emerged relating to (1) friendship and social opportunity (dark green dots), (2) social comparison and gender (light blue dots), (3) motivations to be active (yellow dots) and (4) contextual factors (red dots). Three of the groups comprised of one excerpt only (ID1, ID36 and ID19). These excerpts appeared to cut across a number of other themes and as a result were not consistently sorted by YAP members.

### Theme 1: Friendship and social opportunity (dark green)

This grouping included excerpts relating to the importance of friendship networks in providing both the opportunity and context for physical activity:

*‘Cause if you’re walking to school by yourself it’ll take forever but when you’re with your mates like cause yeah they’re talking about stuff it seems really quick so you could just like walk for ages and you wouldn’t even know’. (ID8)*

Using closeness centrality to identify the most central topics within each cluster, results indicate ID7, ID8 and ID9 as all equivalently central. Qualitative interpretation supports this finding, as each excerpt specifically addresses the importance of friendship in relation to engaging in physical activity.

Participants also appeared to feel more comfortable being active in groups as a means of passing the time and avoiding unwanted social stigma or labelling. As one excerpt suggested:

*‘I’m going to be scundered (embarrassed) walking about alone but when you have your friend with you…you’d be more encouraged to do more walking…’. (ID7)*

Excerpt ID9, further reinforced this opinion, *‘it would have been easier if we were…with our friends’.* The other two excerpts suggest a more competitive role played by friendship networks, explaining their more peripheral role within the cluster. *‘Yeah I was just like competing with myself (in the summer) but….if we were still in school I would’ve been like I need to beat her’ (ID4)* and *‘you’re just trying to beat your friends’ (ID3).* Nonetheless, the data here illustrates how the inclusion of others provided the appropriate context for physical activity to occur.

### Theme 2: Social comparison and gender (light blue)

Social Comparison Theory suggests that individuals are propelled to improve their performance and ‘simultaneously minimize or pre-empt discrepancies between their and other persons’ [37]. As such, the competition-based element of the StepSmart Challenge was designed to capitalise on this process. For female participants, however, this was shown to be a barrier to participation. This grouping revealed that by not separating the active and less active participants, the attraction of the competition diminished, as it was not viewed as ‘a level playing field’. One participant noted, *‘you knew you probably weren’t going to win so…there’s really no point in wearing it’ (ID34).* Closeness centrality data further supports this conclusion with ID34 *r*ated the most central excerpt. This is visually represented on Fig. 4 with ID34 showing the shortest paths between other nodes within the cluster.

In general, female participants were defeatist when making comparisons with those who they perceived to be more active, *‘you know (Name) gonna get it, she’s going to win it! Nobody tries cos (Name) gets it every week’ (ID32)* and *‘whenever you found out that you’re actually losing there’s just no point’ (ID2).* However, these sentiments were not reflected in excerpts relating to male participants who took in the StepSmart Challenge. For example, under theme 1 (friendship and social opportunity) social comparison was suggested as a motivating rather than de-motivating factor, *‘you’re just trying to beat your friends’ (ID3)*. This excerpt is particularly interesting because it was sorted with excerpts that were allocated to several different groups.

Social comparison has been linked with friendship relatedness whereby individuals appear to be more competitive against those from within their own social network [38, 39]. This suggests that the absence of this component may have been an important factor regarding overall engagement by female participants. As such, social comparison in the context of this thematic cluster, was viewed negatively.

### Theme 3: Motivations to be active (yellow)

Closely aligned with the behaviour-change literature [22] the excerpts included in the largest grouping related to the benefits of setting targets and being able to monitor progress using the pedometers. One excerpt stated, *‘because you look at it and you’ve only took like 5000 (steps); you realise you have to take ten so you just try to push yourself to do it, to try reaching it’ (ID15).* The relevance of this is further demonstrated in Fig. 4 whereby excerpts relating specifically to monitoring and goal setting are placed at the centre of the grouping, ‘*cause if you had one (pedometer) in training like I was looking at myself as being like I need to train more to get these up and then I did it’ (ID26)* and ‘*it wasn’t about the competition it was just about seeing how many steps you took’ (ID5).*

This grouping also tended to include excerpts denoting a level of intrinsic motivation (inherently interesting or enjoyable). One participant noted, *‘like just get up and do it and then when you do it like it actually makes you feel better so it just makes you want to do more of it’ (ID22).* This suggests that StepSmart was beginning to move participants away from extrinsically motivated behaviours (something because it leads to a separable outcome) [40]. Moreover, ID22 recorded the highest betweenness centrality score within the cluster suggesting that it links some of the subthemes.

Participants also explored new ways to become active as means of achieving goals. One participant noted, *‘(I was) just being lazy sitting in the house and then instead of sitting down I was just walking about the house even cause there’s nowhere really round my way so I just walk around the house like up and down the stairs and stuff’ (ID10).*

In summary, participants found the ability to set goals and monitor progress beneficial. Those who reported these experiences also appeared to be less motivated by winning prizes and revealed more intrinsically motivated reasons for being active.

### Theme 4: Contextual factors (red)

The final grouping relates mainly to the opportunities (or lack thereof) for physical activity. This grouping is of particular interest given that it was not identified as a major theme during researcher-led thematic analysis. Within this group, ID30 achieved the highest closeness centrality score, and is the only node directly attached to ID35. Qualitative interpretation suggests that while ID27, ID28 and ID29 are closer aligned thematically, ID30 is central to the formation of the cluster, acting a bridge between other nodes. The school day was viewed as both a facilitator, e.g. ‘*school is better about the structure part but the summer is better cause you could use it (pedometer) more and you were out more’ (ID29)* by providing structure, and as a barrier by being exhausting and restricting movement:

*‘Cause when you go to school and you come home and you’re like drained and all and you have to go your homework but in the summer its different cause you don’t have to go to school so you don’t be as tired and you can get more sleep as well’. (ID28)*

Other excerpts suggested that there would be a greater chance of getting a higher number of steps during the summer phase of StepSmart, ‘*I reckon the summer (competition)… will be higher than it was in school’(ID27) and ‘yeah cause you got more time to spend to do stuff’ (ID30).* Again there was some separation between excerpts contained with this grouping. ID35 appears a potential outlier with little relevance to other excerpts contained within the grouping. This is demonstrated by the thinness of the line attaching it to the group as well as closer inspection of the excerpt itself.

### Miscellaneous excerpts

ID1, ID36 and ID19 did not fall within any of the groups following PTE analysis. This indicates that YAP members had different perspectives about where to sort these quotes, which can be seen in Fig. 4 by the similarity of line thickness attaching the relevant nodes to nodes in multiple groups. That is, these excerpts are perceived to be similar to excerpts relating to one theme by some sorters, and to a different theme by other sorters.

For example, ID1 *(‘I was getting like really mad at (name) like trying to beat her… she’s doing like 20,000 (steps) a day now or something’*) was sorted with ID32 *(‘you know (Name) gonna get it, she’s going to win it! Nobody tries cos (Name) gets it every week’)* by three YAP members. While both involve an element of social comparison, ID32 has a more negative tone which relates more to other excerpts within theme 2 and thus may partially explain the separation between them. ID19 is most closely connected to group/theme 3 (motivations to be active). This appears logical given the fact that it specifically mentions incentives, *‘could be…class prizes instead of individual ones’ (ID19).* However, as the excerpt is ambiguous and lacks a clear statement of intent, YAP members appear to have struggled to consistently sort this with other incentive excerpts. Finally, the negative connotation contained within ID36 *(‘you didn’t know what progress you were making so it wasn’t really any benefit’*) may have resulted in its close proximity to nodes within group/theme 2 (social comparison and gender). However, the content could also be interpreted under group/theme 3 (motivations to be active) and thus may explain why it was inconsistently sorted by YAP members.

Table 2 highlights potential disagreements or differing interpretations among YAP members. This table shows the total number of piles created by YAP members and the number of groupings that were preserved within the final model. A preserved pile is one where all the excerpts contained in the pile (of that sorter) are also in the same group assigned by the clustering algorithm. The overall low preservation rate is partly because any pile that included one of the inconsistent excerpts (ID1, ID19 and ID36) could not be preserved. From Table 2, P5 had the lowest number of entries (*n* = 2) preserved within the final model. This suggests that P5 had a different perspective on the qualitative data that may be appropriate to follow up, perhaps with an interview.

**Table 2:** Preservation of excerpts by youth advisory panel (YAP) participants

Participant	Piles	Piles with two or more excerpts	Piles preserved
P1	9	8	4
P2	7	7	5
P3	8	8	3
P4	6	6	3
P5	7	7	2
P6	7	7	5
P7	14	14	9
P8	9	9	5

## Discussion

The aim of this proof-of-concept study was to investigate the feasibility of a novel approach to participatory data analyses and interpretation (PTE). The most effective approaches to involving children and young people in data analysis remains debated within the academic literature. While some advocate comprehensive training in data analysis techniques [19] others suggest that this ‘defeats the purpose’ of user involvement and young people should be encouraged to develop their ‘own ways of exploring their lives’ [41]. Moreover, promising existing techniques, such as the Voice Relational Method [13], are resource intensive and perhaps not suited within large-scale interventions or to those less experienced in qualitative research.

The purpose of PTE is to address some of these issues by providing a robust, accessible and reproducible approach to meaningful end-user involvement in data analyses and interpretation. Informal feedback taken immediately after the sorting task revealed that this process was easy to understand and largely accessible for YAP members. While the YAP included a range of mixed-ability pupils, all members completed the activity within the allotted time and with little assistance from the research staff present. PTE has been designed to be accessible to both researchers and participants/co-researchers while minimising the influence of researcher bias.

An important aspect of PTE is the ability to visually identify emerging themes in order to gain a better understanding of the relationship between them. Similar research by Prior and colleagues ([42]: p. 76) highlights the benefits of network diagrams for qualitative analyses, noting that it ‘enables the reader to get a clear sense of the relative importance of any given issue in a wider context, and how strongly or weakly that issue connects to others in the web (network)’. As such, PTE appears both practical and meaningful by showing how community detection algorithms and other network methods can inform qualitative investigation. It enabled the research team to identify themes, central concepts within those themes, and excerpts that cut across a number of different themes (as seen within three of the PTE groupings), all of which enriched the quality of the analysis.

### Comparison with researcher-led analysis

PTE findings were similar to the researcher-led thematic analysis. Detailed elsewhere [27], the themes identified in the conventional thematic analysis concern incentives, competition and the influence of friends in motivating physical activity. These can be broadly mapped to the PTE groups of *motivations to be active, social comparison and gender, and friendship and social opportunity*, respectively. This process was, therefore, particularly useful in confirming some of the findings from the researcher-led analysis. As such, PTE may provide a robust process which to challenge and confirm the conventional analysis, increasing the overall validity of findings.

Beyond this broad similarity, PTE analysis also prompted additional insight. One of the most interestingly findings from PTE analysis was the complex and multifaceted nature of team-based competition, particularly in relation to female pupils. In accord with the thematic analysis [27], PTE revealed social networks and goal setting as important contributing factors regarding continued engagement with the StepSmart Challenge. However, there were some discrepancies that require further exploration. For example, PTE groupings presented goal setting as taking place predominantly on a personal level (i.e. the setting of individual targets rather than targets likely to win prizes) whereas conventional thematic analysis [27] framed this process largely within the context of the pedometer competition. While both appear plausible, PTE points to a more intrinsically motivated behaviour pattern in relation to goal setting. One study by Routen and colleagues [43] found that individualised goals were more likely to support increases in moderate-to-vigorous physical activity than group-based goals. This is interesting given what we learned from focus groups in relation to peer influence. However it may indicate the continued importance of personal and intrinsic goals as a key element for sustained change [44].

It is also important to note that *contextual factors* represent an additional grouping identified through PTE that was not highlighted as a major theme or subtheme during researcher-led thematic analysis [27]. This is perhaps indicative of the additional value of PTE by providing ‘insider’ knowledge [24]. PTE highlighted the structure of the school day as a barrier to physical activity. Findings suggest that fatigue was also an important factor which influenced participant’s decision(s) and motivation to be active when the school day was complete. As such, physical activity incorporated throughout the school day (e.g. during the daily commute, during break times or ‘active’ classrooms) might be an appropriate option for young people. Increasing pressures related to school work and social activities may be squeezing out time dedicated to physical activity, particularly if one’s friends are not active. However, it is important to note that the identification of this additional theme may have also been due to the inclusion of young people’s perspective rather than the PTE method alone. A core aim of PTE is to facilitate young people’s perspective in the interpretation of research findings and so it is not possible to disentangle these two processes.

As this is a proof-of-concept study, further development of the method will be required using different data sources and populations. It is also important to further embed this method within a participatory process and, as such, the process described above may be revised and expanded to include the following: (1) in-depth discussion with YAP members regarding PTE output and associated themes within each group; (2) discussions with YAP members regarding the overall acceptability of the method by more diverse groups; and (3) feedback from YAP members regarding the placement order of the excerpts and the pile diversity. Data relating to the preservation of excerpts within the final model (Table 2) may also be useful for guiding qualitative discussion to identify those with differing opinions and interpretations.

### Limitations

There are some limitations to this approach which will be addressed in future research. In particular, PTE was devised as a proof-of-concept study within an evaluation and further work is required to validate the method in a variety of settings.

Some of the researchers who were involved in focus group data collection also selected the excerpts given to YAP. While this took place before any formal analysis had been undertaken, there is still the possibility of selection bias. Additional qualitative work would have been useful to further explore some of the novel insights and discrepancies with the researcher-led thematic analysis. YAP members could have been presented with the PTE groupings for discussion and asked to name the themes themselves. This would have reduced researcher bias.

While feedback on acceptability and usefulness of the grouping task was collected informally after the grouping activity had taken place, the authors recognise that an additional formal meeting or focus group with YAP members following PTE analysis would have been useful. This is important as the network analysis methods employed within PTE only produce groupings and not themes (which need additional interpretation and analysis). Another possible limitation was that the number of excerpts given to YAP members was limited to 40. This was due mainly to practical considerations centring on the timetables of the schools involved. The time allotted (2 h) was viewed as sufficient to perform the group task. However, any increase in the total number of excerpts may require additional sessions. It is also considered that the number of sorters could be increased to further strengthen the validity of findings in future work. While sample size calculation within qualitative research is fairly new (see Fugard and Potts [45]) and somewhat contentious, it might be difficult to claim ‘data saturation’ using only 40 excerpts. Finally, the measure of conceptual distance and betweenness employed in the community detection algorithm should be further tested by comparing the thematic groups obtained with alternative algorithms. Nonetheless, as a proof of concept, and with preliminary findings supported by researcher-led analysis, using a PTE method is a promising approach to support meaningful research coproduction.

## Future research

In order to develop the method further, future research should consider greater involvement of users (both during the initial selection of excerpts and following the generation of groupings). One must also investigate what an ‘appropriate’ (i.e. not burdensome for the young people) and ‘sufficient’ (i.e. able to reach data saturation or ensure that core themes are not missed) number of excerpts from the transcripts might be. However, this may vary by population and focus group topic. Further areas of investigation include, the number of sorters, the number of excerpts, the training time needed for co-researchers and the use of alternative algorithms to produce groupings.

## Conclusion

This study describes a promising approach to participatory data analyses and interpretation. Proof of concept has been demonstrated through the ability of thematic patterns to be generated from PTE groupings. Moreover, recruitment, retention and training of YAP members appeared successful. The authors are, however, aware that reducing qualitative research to a list of technical procedures is overly prescriptive and can result in ‘the tail wagging the dog’ [46]. As such, some consensus-building exercises with qualitative researchers and those involved in RCT-based research will be considered. PTE is designed to assist the end user in bringing their ‘lived experience’ to the analyses process. Through further development and testing, it is hoped that researchers will find it easier to involve end users in the data analyses process using PTE. Beyond its potential as a structured, transparent and reliable method of participatory data analyses, a strength of PTE is that its implementation requires little prior knowledge or specialist skills on behalf of users or researchers. Furthermore, it may reduce the capacity for researcher bias by producing user-generated data in which to support or challenge emerging narratives. For those engaged in trial-based research or who are less au fait with participatory data analysis, the accessibility of PTE will be of particular interest.

## Appendix

**Table 3:** Full list of excerpts given to youth advisory panel (YAP) members

Number	Excerpt text	Central
Friendship and social opportunity		
ID3	You’re just trying to beat your friends.	
ID4	Yeah I was just like competing with myself but like if there was like if we were still in school I would’ve been like ‘I need to beat her.’	
ID7	Makes you feel like I’m going to be scundered walking about alone but when you have your friend with you like you’d be more encouraged to do more walking if you’re like walking with your friend.	Yes
ID8	Cause if you’re walking to school by yourself it’ll take forever but when you’re with your mates like cause yeah they’re talking about stuff it seems really quick so you could just like walk for ages and you wouldn’t even know.	Yes
ID9	It would have been easier if we were like with our friends.	Yes
Social comparison and gender		
ID2	Whenever you found out that you’re actually losing there’s just no point.	
ID14	I would like to be in her team cos then I wouldn’t have to do work.	
ID32	You know (Name) gonna get it, she’s going to win it! Nobody tries cos (Name) gets it every week.	
ID33	There is really no point because see at the end like I am literally only going to do it if you actually do change the PE gear.	
ID34	It’s just sort of cause you knew you probably weren’t going to win so you’re just like ‘there’s really no point in wearing it’.	Yes
Motivations to be active		
ID5	It wasn’t about the competition it was just about seeing how many steps you took.	
ID6	Yeah you didn’t want to let your team down.	
ID10	Just being lazy sitting in the house and then instead of sitting down I was just walking about the house even cause there’s nowhere really round my way so I just walk around the house like up and down the stairs and stuff.	
ID11	Yeah like even like walking more instead of getting your mummy to drop you to places like you just walk down like if it’s not even far away.	
ID12	Just healthier in general I think, cause I don’t think if you went out and bought one you’d be getting competitions and winning and stuff; think you’ll just be getting fitter.	
ID13	Like girls want to look more good than just about the competition, like girls will always (care) about their appearance so if you said make them healthier.	
ID15	Because you look at it and you’ve only took like 5000 (steps); you realise you have to take ten so you just try to push yourself to do it, to try reaching it.	
ID16	I want to be more active cos if you see your score’s quite low it’s just… You feel kinda bad as everyone else is like really high and actually try and do this.	
ID17	I don’t want to grow up to be like a wee lazy person who doesn’t do anything!	
ID18	It was quite interesting like, how many steps you’re doing.	
ID20	Maybe before that I like I would’ve been way more active but then it just sort of stopped cause I wasn’t as motivated and then like the pedometers would’ve just like started me up again to be competitive.	
ID21	I’d still do it anyway if there wasn’t any prizes.	
ID22	Like just get up and do it and then when you do it like it actually makes you feel better so it just makes you want to do more of it.	Yes
ID23	You can set yourself like goals and targets and then try to beat them each day.	
ID24	It shows you how much like steps you can take in a day, like you wouldn’t even think you were walking that much.	
ID25	The app was helpful. It worked for me, it worked really well for me.	
ID26	Cause if you had one in training like I was looking at myself as being like I need to train more to get these up and then I did it.	
ID31	You do get like a lot of exercise when walking all around school and all upstairs and going down.	
ID37	It’s not even about like trying to be skinny it’s just about being healthy for me.	
ID38	It’s just the prizes work cos then you really want to win them and get more competitive.	
ID39	You’ll become healthier and fitter if you work with it.	
ID40	Cause you saw that they weren’t just like wee rubbish prizes they were really good ones.	
Contextual factors		
ID27	I reckon the summer one will be higher than it was in school.	
ID28	Cause when you go to school and you come home and you’re like drained and all and you have to go your homework but in the summer its different cause you don’t have to go to school so you don’t be as tired and you can get more sleep as well.	
ID29	School is better about the structure part but the summer is better cause you could use it more and you were out more.	
ID30	Yeah cause you got more time to spend to do stuff.	Yes
ID35	And the problem is should be like every so often check whose lost it and then ask them ‘do you really want another one to continue’ because some people might not want to another one to continue, they are being given it and then they’ll lose that one as well.	
Unassigned		
ID1	I was getting like really mad at (Name) like trying to beat her… she’s doing like 20,000 a day now or something.	
ID19	Could be like class prizes instead of individual ones.	
ID36	You didn’t know what progress you were making so it wasn’t really any benefit.	

## Abbreviations

PPI: Public and Patient Involvement
PTE: Participatory Theme Elicitation
YAP: Youth Advisory Panel
